# Supplementary material for: High-throughput identification of prefusion-stabilizing mutations in SARS-CoV-2 spike
Source: Nat Commun. 2023 Apr 10;14:2003. doi: 10.1038/s41467-023-37786-1 (PMC10086000; doi:10.1038/s41467-023-37786-1)
Supplement: Supplementary file 1 — Supplementary Information [file 41467_2023_37786_MOESM1_ESM.pdf]

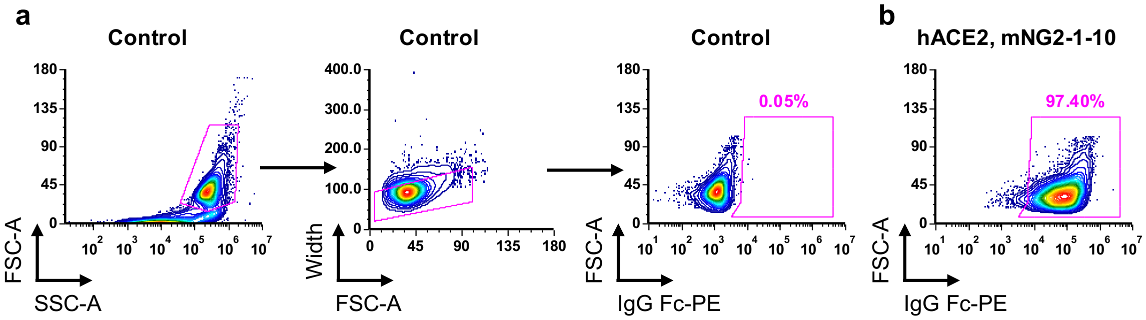

Fig. S1 | Validation of hACE2, mNG2$_{1\text{-}10}$-expressing cell line. a,b, Flow cytometry plots showing **(a)** untransfected control and gating strategy in this verification experiment, and **(b)** surface expression of hACE2 in HEK293T landing pad cells. Primary antibody used was S RBD with an Fc tag.

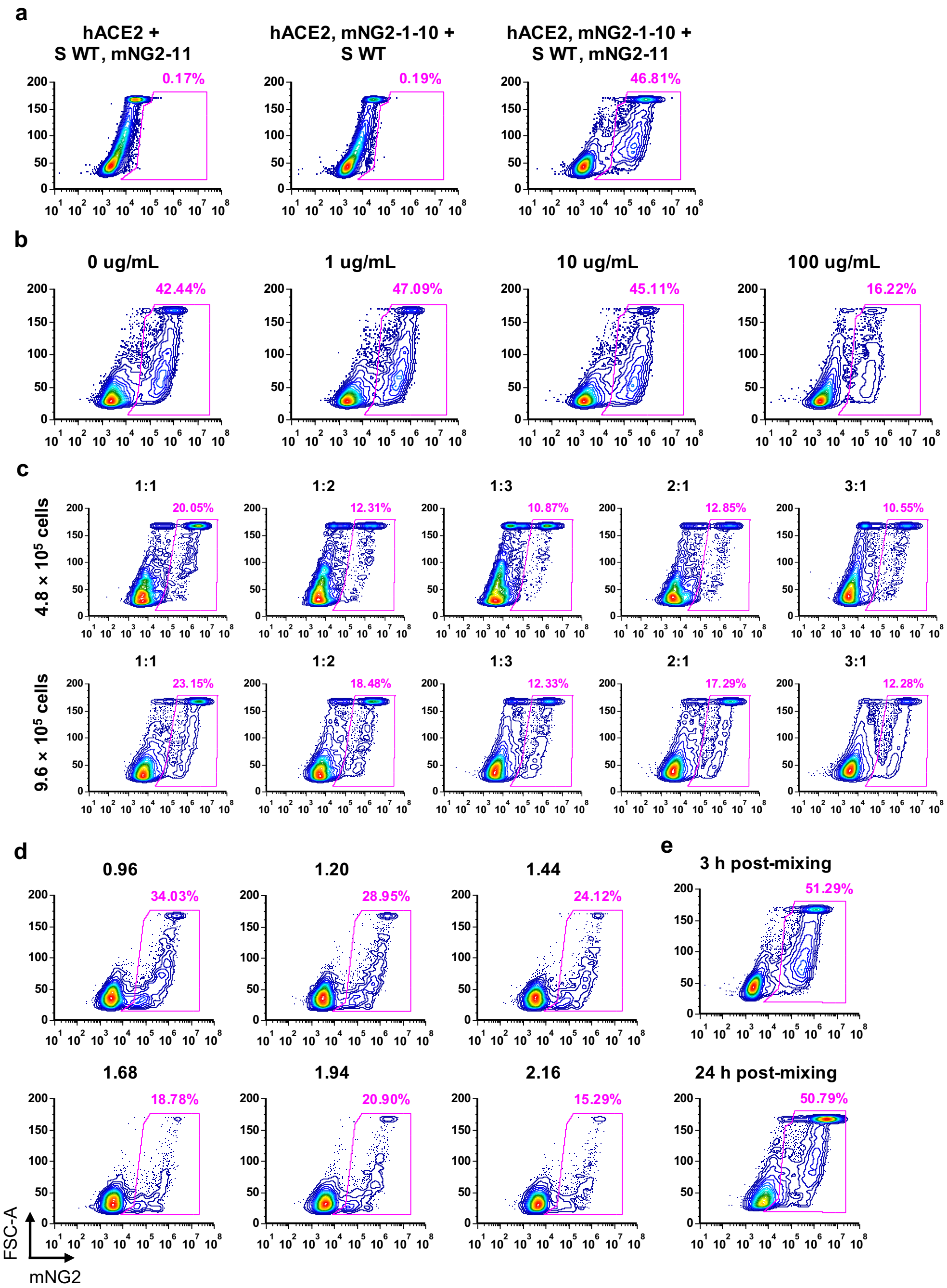

Fig. S2 | Validation and optimization of fusion assay. a, Flow cytometry plots of fusion assay between hACE2- and S-expressing cells showing detectable green fluorescence when both mNG2$_{1-10}$ and mNG2$_{11}$ were expressed, and negligible fluorescent background signal when either mNG2$_{1-10}$ or mNG2$_{11}$ was expressed. **b,** Addition of CC40.8[1], a neutralizing antibody targeting the stem helix, to S, mNG2$_{11}$-expressing cells one hour prior to mixing with hACE2, mNG2$_{1-10}$-expressing cells decreased fusion events. Antibody concentration is indicated above each plot. **c,** Optimization of the ratio between hACE2, mNG2$_{1-10}$- and S, mNG2$_{11}$-expressing cells for fusion

assay. Total cell number in co-culture is indicated, and the ratio of hACE2, $mNG2_{1-10}$- to S, $mNG2_{11}$-expressing cells is shown above each plot. **d,** Optimization of total cell numbers of hACE2, $mNG2_{1-10}$- and S, $mNG2_{11}$-expressing cells for fusion assay. Total cell number, in millions, of hACE2, $mNG2_{1-10}$- and S, $mNG2_{11}$-expressing cells in co-culture is shown above each plot. Equal numbers of hACE2, $mNG2_{1-10}$- and S, $mNG2_{11}$-expressing cells were co-cultured for 3 hours. **e,** Optimization of time point for fusion sorting. Equal numbers of hACE2, $mNG2_{1-10}$- and S, $mNG2_{11}$-expressing cells totaling 5.0×10^5 cells/mL were mixed and analyzed via flow cytometry at the indicated time point. Fig. S11b shows the gating strategy for optimization experiments.

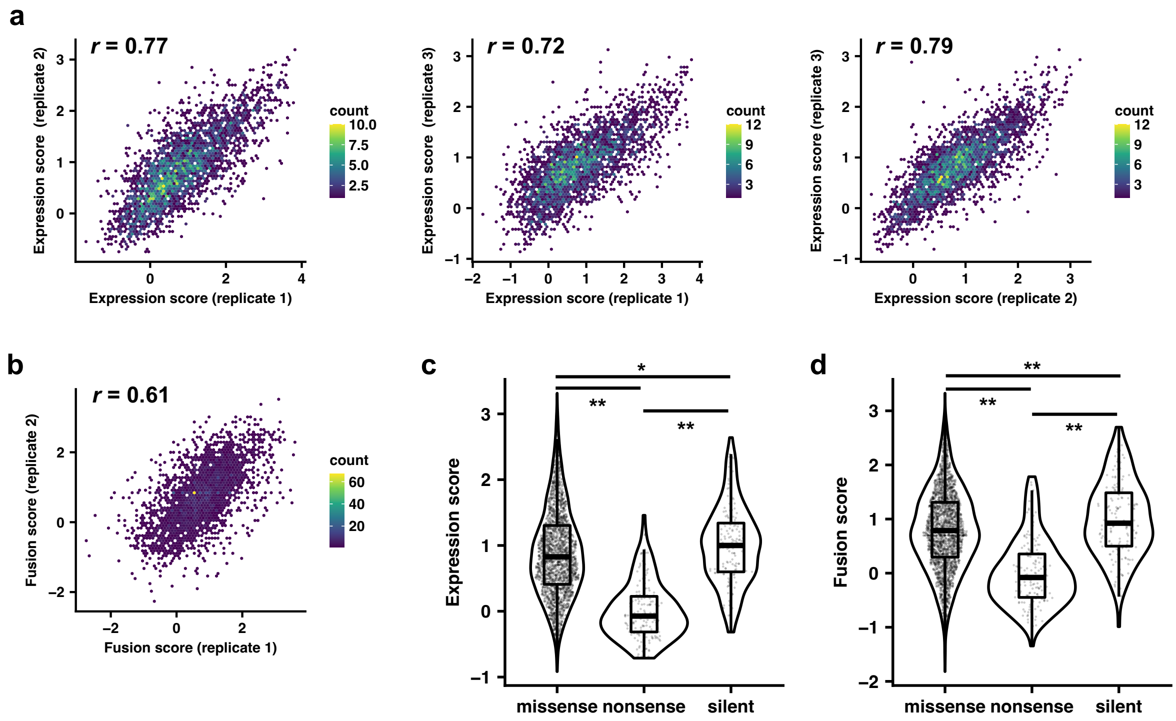

Fig. S3 | Additional analyses for expression and fusion assays. a,b, Correlation of expression scores **(a)** and fusion scores **(b)** between replicates. Pearson correlation coefficient, *r*, is shown for each plot. **c,d,** Violin plots of expression scores **(c)** and fusion scores **(d)** of missense, nonsense, and silent mutations are compared. Box-and-whisker plots are also shown. The center corresponds to the mean, bounds of box represent first and third quartiles, and whiskers extend to the 5th and 95th percentiles. *, $p < 0.05$; **, $p < 0.001$. Exact p-values from two-sided t tests are shown in Table S6. Expression and fusion scores are averages from $n = 2$ independent biological replicates. Data are plotted from Supplementary Data 1.

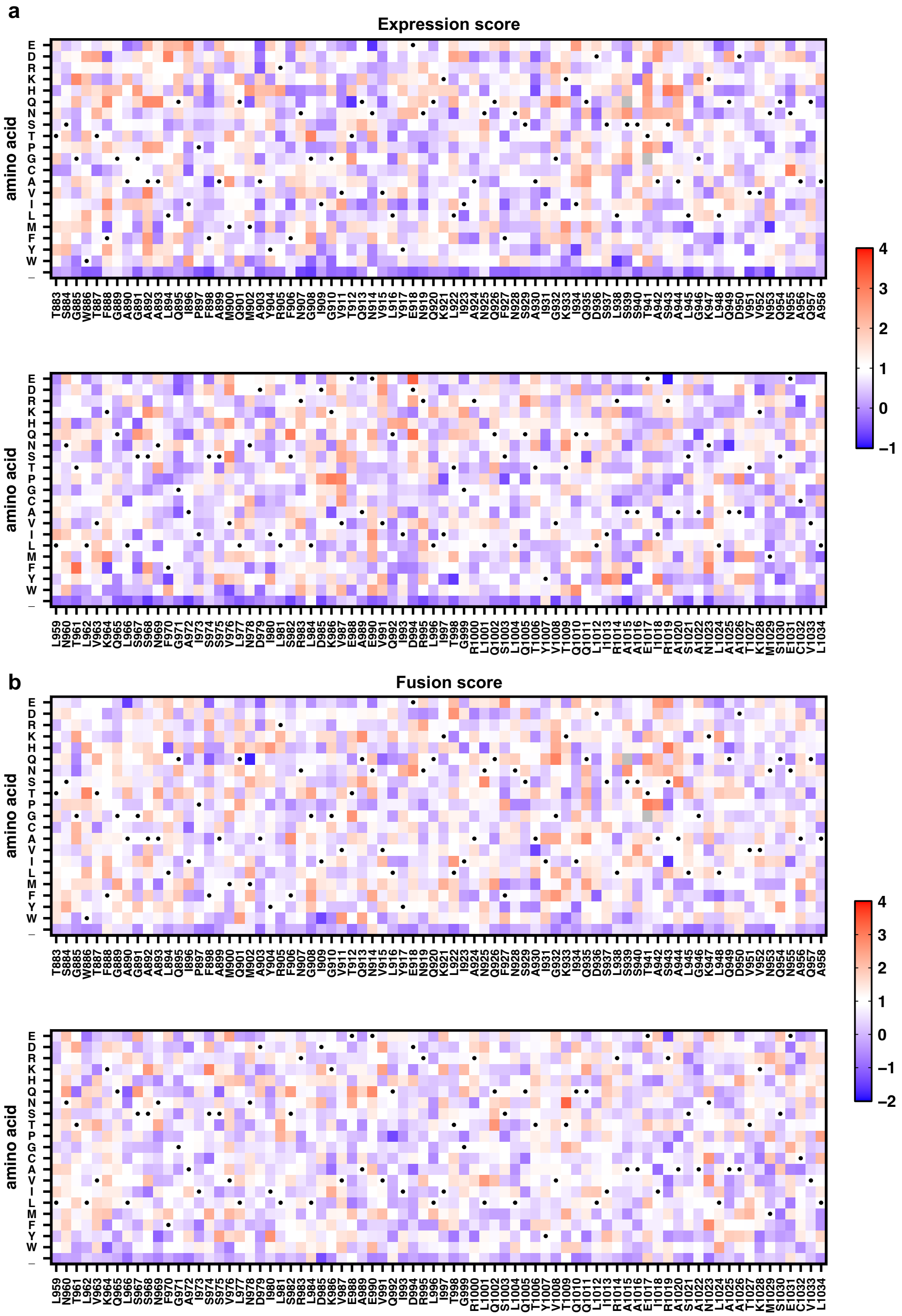

Fig. S4 | Heatmaps of expression and fusion scores. a-b, Expression scores **(a)**, and fusion scores **(b)** of all amino-acid mutations in the DMS library. Underscore (_) in amino acid represents a nonsense mutation. Dots indicate WT. Grey squares indicate mutations that were not observed. Expression and fusion scores shown are the mean of n = 3 and n = 2 independent replicates, respectively. Lower expression score implies lower surface expression of the S protein, whereas

lower fusion score suggests fusogenicity is impaired. Abbreviations of amino acid residues are as follows: A, alanine; C, cysteine; D, aspartic acid; E, glutamic acid; F, phenylalanine; G, glycine; H, histidine; I, isoleucine; K, lysine; L, leucine; M, methionine; N, asparagine; P, proline; Q, glutamine; R, arginine; S, serine; T, threonine; V, valine; W, tryptophan; Y, tyrosine. Data are plotted from Supplementary Data 1.

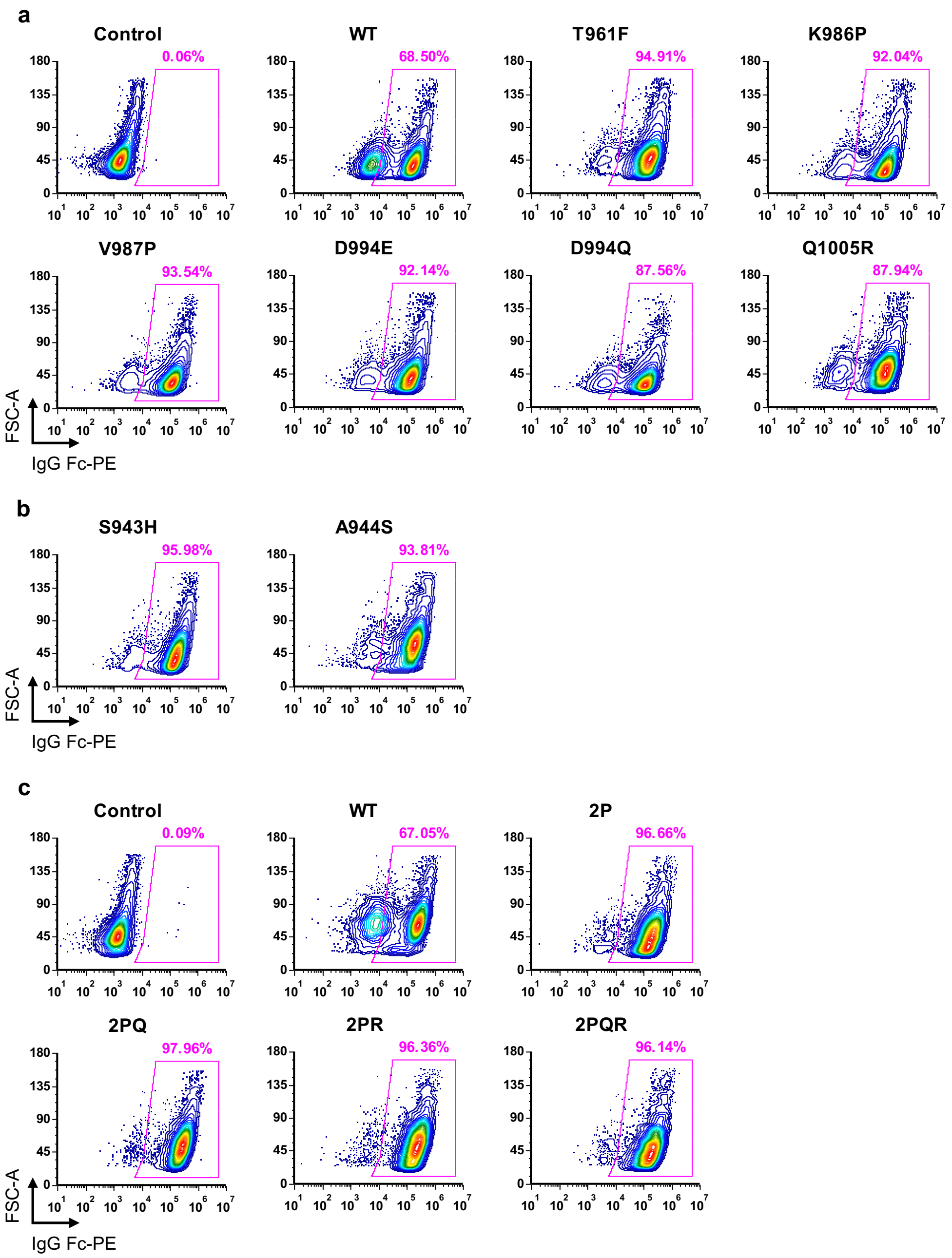

Fig. S5 | Validation of surface expression of mutants of interest. a-c, Flow cytometry plots showing surface expression of candidate prefusion-stabilizing mutations **(a)**, fusion-enhancing mutations **(b)**, and combinations of candidate prefusion-stabilizing mutations **(c)**. Plots are representative of *n* = 3 independent replicates. Abbreviations for combinatorial mutations are as follows: 2P, K986P/V987P; 2PQ, K986P/V987P/D994Q; 2PR, K986P/V987P/Q1005R; 2PQR, K986P/V987P/D994Q/Q1005R. Fig. S11a shows the gating strategy for this experiment.

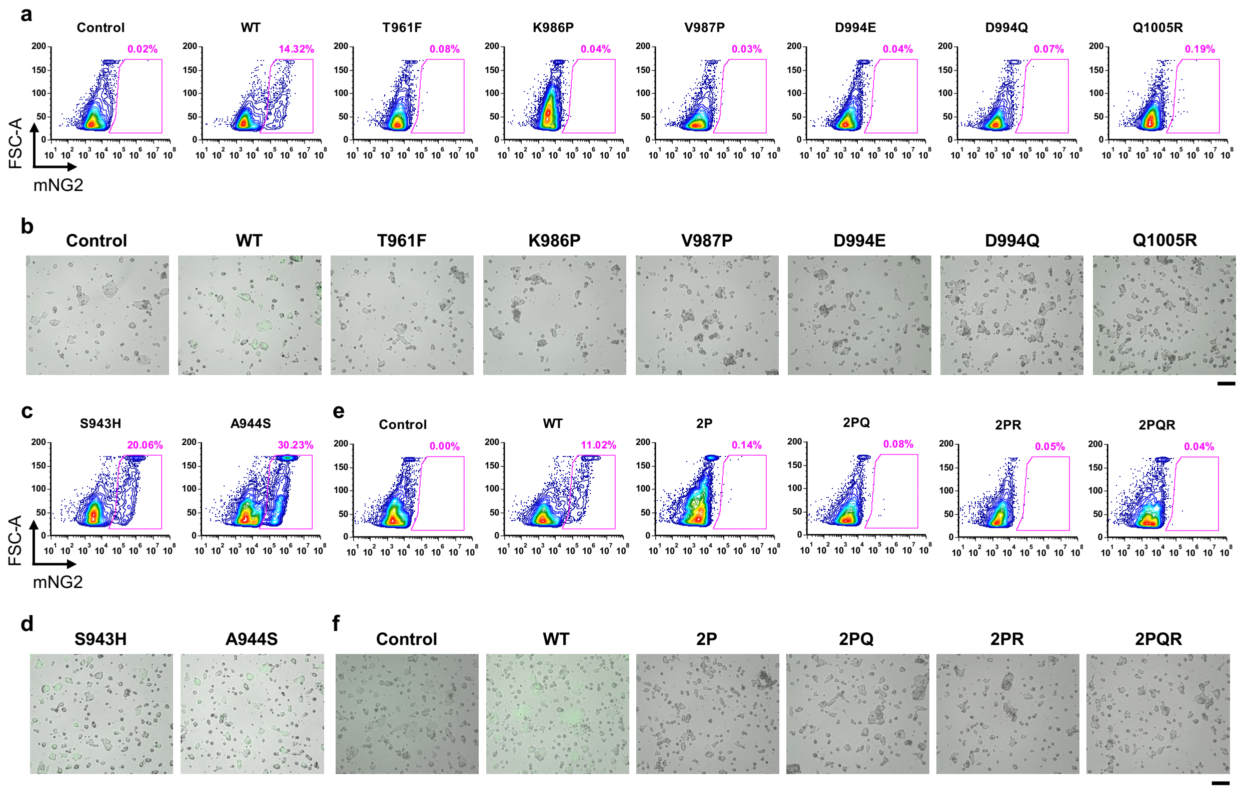

Fig. S6 | Fusion activity of mutants of interest. a,b, Flow cytometry plots **(a)** and micrographs **(b)** of S-expressing cells with fusion-incompetent mutations at 3 hours post-mixing with hACE2-expressing cells. **c,d,** Flow cytometry plots **(c)** and micrographs **(d)** of S-expression cells with fusion-enhancing mutations at 3 post-mixing with hACE2-expressing cells. **e,f,** Flow cytometry plots **(e)** and micrographs **(f)** of S-expression cells with combinations of fusion-incompetent mutations at 3 hours post-mixing with hACE2-expressing cells. Plots and micrographs are representative of *n* = 2 independent replicates. Abbreviations of combinatorial mutations are as follows: 2P, K986P/V987P; 2PQ, K986P/V987P/D994Q; 2PR, K986P/V987P/Q1005R; 2PQR, K986P/V987P/D994Q/Q1005R. Scale bar: 100 µm. Fig. S11b shows the gating strategy for this experiment.

a

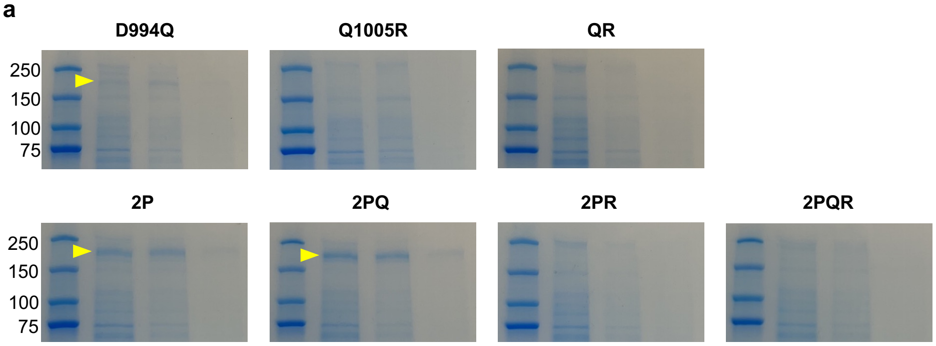

b

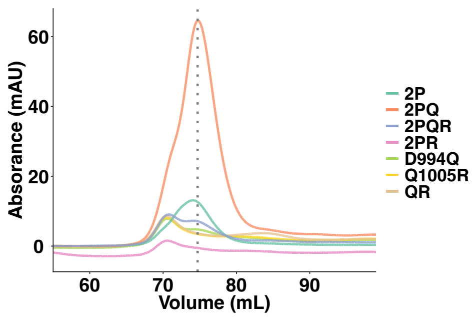

c

Mutant	Culture volume (mL)	Yield (μg per mL of culture)
D994Q	25	0.69
Q1005R	25	Undetectable
QR	25	Undetectable
2P	100	0.66
2PQ	100	2.24
2PR	25	0.05
2PQR	200	0.18

Fig. S7 | Purification of S ectodomain mutants. a, SDS-PAGE gels of soluble spike proteins after His-tag affinity purification. Molecular weights (in kDa) of protein standards are shown. Arrowheads point to the band corresponding to the size of a glycosylated S monomer (~180 kDa). Gels are representative of *n* = 2 independent biological experiments. **b,** Chromatograms of soluble spike ectodomain mutants for biophysical characterization. Dotted line indicates the peak volume (~74 mL) corresponding to the size of a glycosylated spike trimer (~540 kDa). Fractions from 73 mL to 79 mL were collected and concentrated. **c,** Culture volume and yield for each mutant in **b**. Abbreviations for combinatorial mutations are as follows: QR, D994Q/Q1005R; 2P, K986P/V987P; 2PQ, K986P/V987P/D994Q; 2PR, K986P/V987P/Q1005R; 2PQR, K986P/V987P/D994Q/Q1005R. Source data are provided as a Source Data file.

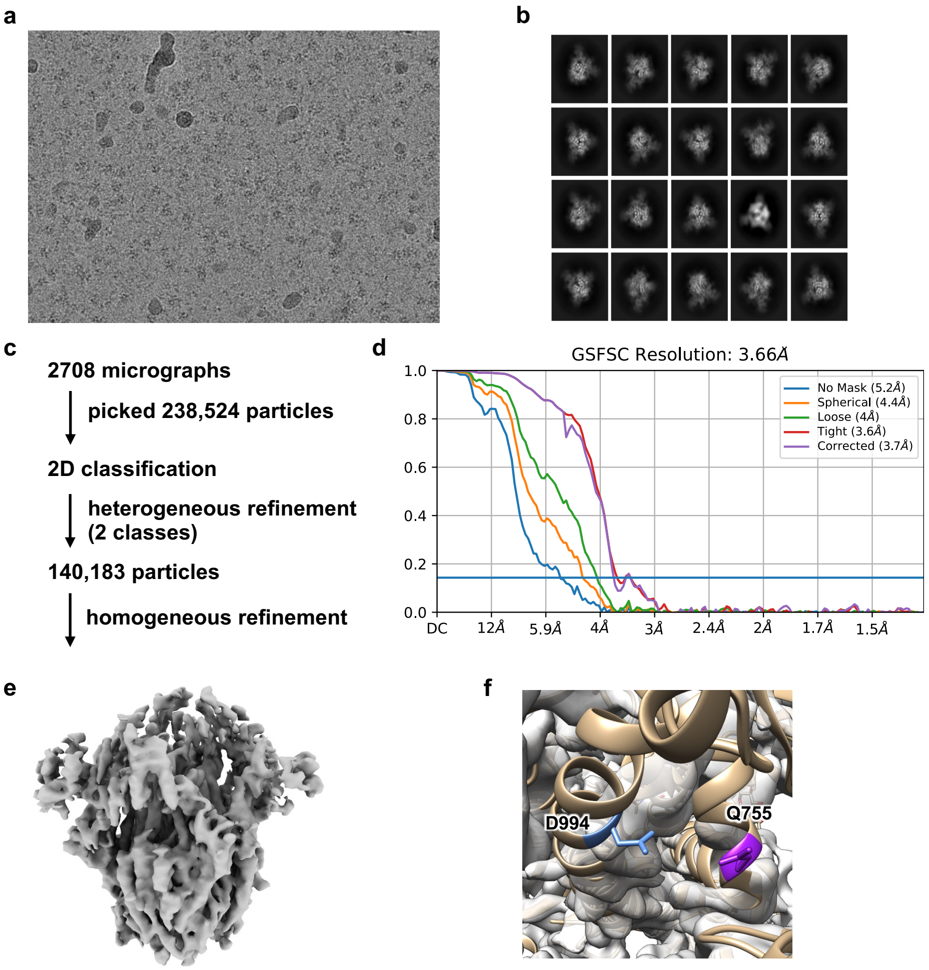

Fig. S8 | Cryo-EM structure validation and analysis. a, Representative micrograph for cryo-EM experiments. **b,** Exemplary 2D classes of soluble S with 2PQ mutations. **c,** Cryo-EM data processing workflow. Data collection and analysis was performed on cryoSPARC Live. **d,** Gold-standard Fourier shell correlation curve of soluble 2PQ ectodomain. **e,** Cryo-EM density map of soluble S with 2PQ mutations after homogeneous refinement. **f,** Fit of cryo-EM density map of soluble S with 2PQ mutations (in grey) to resolved structure of S with 2P mutations (PDB: 6VXX[2]). Helices in 2PQ as indicated by the electron density map are closer compared to those in 2P. Contour level of density map was set to 0.2 in UCSF Chimera.

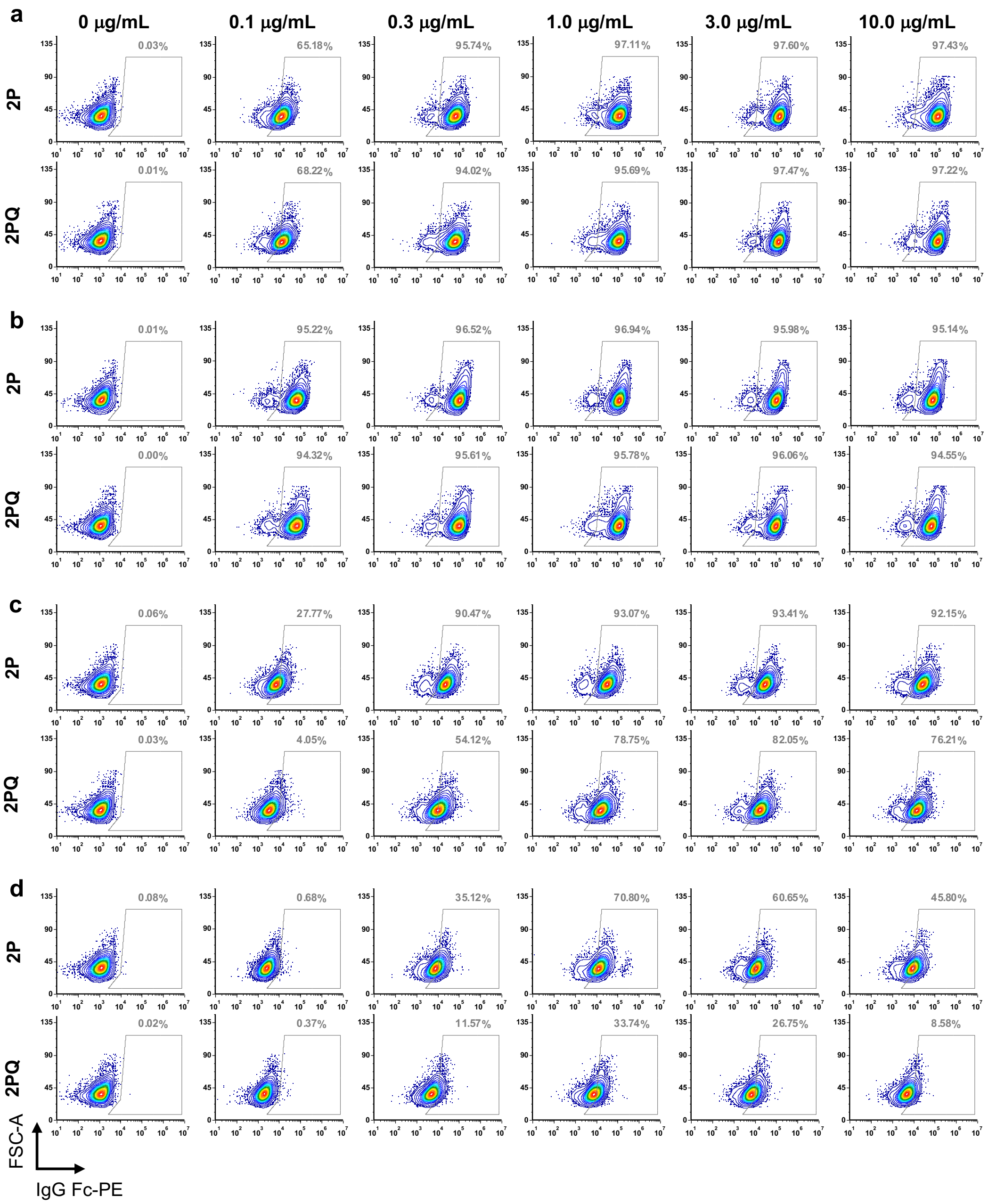

Fig. S9 | Titration of SARS-CoV-2 S antibodies to membrane-bound S 2P and 2PQ. a-d, Flow cytometry plots from titration of membrane-bound S bearing 2P or 2PQ mutations with S2M28[3], an N-terminal domain antibody **(a)**, CC12.3[4], a receptor-binding domain antibody **(b)**, COVA1-07[5], a heptad repeat 1 antibody **(c)**, and CC40.8[1], a stem helix antibody **(d)**. The concentrations of antibodies are indicated. Fig. S11a shows the gating strategy for this experiment.

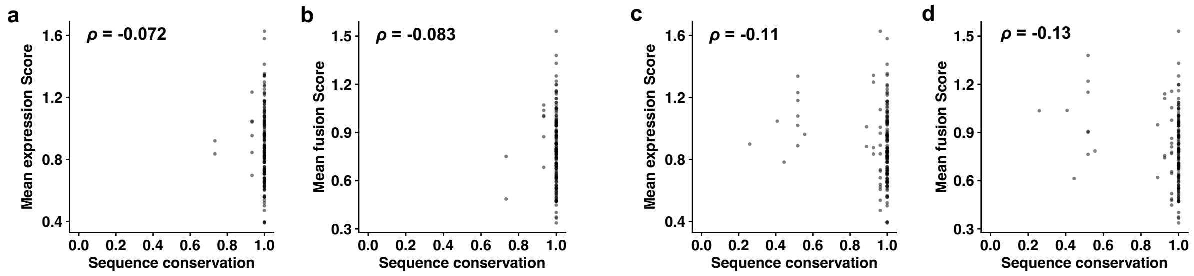

Fig. S10 | Sequence conservation analysis of S2 HR1 and CH mutations. a,b, Plots of mean expression score **(a)** and mean fusion score **(b)** against natural frequency in HR1 and CH of major SARS-CoV-2 variants (**Table S1**). **c,d,** Plots of mean expression score **(c)** and mean fusion score **(d)** against sequence conservation of the S2 HR1 and CH regions of related betacoronaviruses listed in **Table S7**. Spearman correlation coefficients, *ρ*, are shown in **a-d**. Source data are provided as a Source Data file.

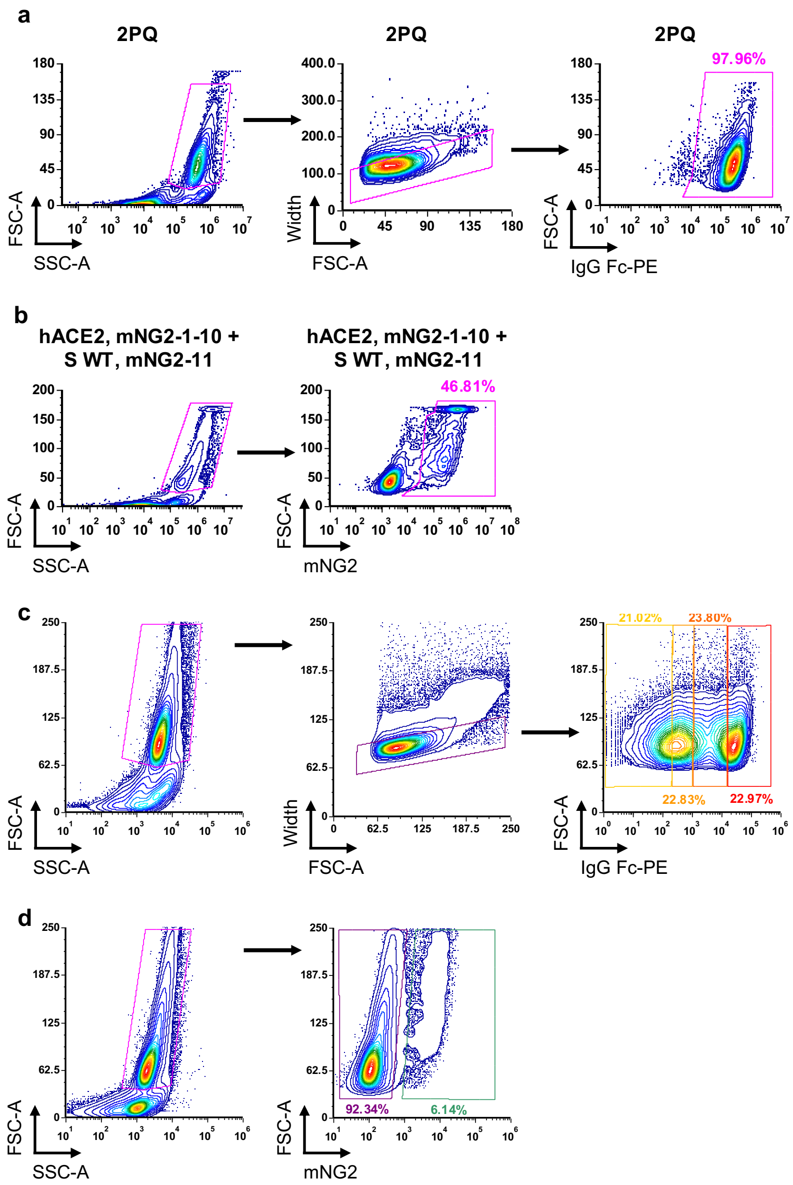

Fig. S11 | Gating strategies for flow cytometry and fluorescence activated cell sorting (FACS). a, Gating strategy used for flow cytometry to assess surface expression of S via PE fluorescence. **b,** Gating strategy used for flow cytometry to determine fusion of hACE2- and S-expressing cells via mNG2 fluorescence. **c,** Gating strategy used for FACS to sort the DMS library of S-expressing cells based on levels of PE fluorescence. **d,** Gating strategy used for FACS to sort the co-culture of the DMS library of S-expressing cells and hACE2-expressing cells at 3 hours post-mixing, based on presence or absence of mNG2 fluorescence.

SUPPLEMENTARY TABLES

Table S1. Mutations that are included in our DMS library (residues 883 to 1034) and also found in SARS-CoV-2 variants of concern and variants of interest.

**Variants of concern**		
**PANGO Lineage**	**Mutations**	**GenBank Reference**
Alpha (B.1.1.7)	S982A	MZ344997.1
Beta (B.1.351)	-	MW598419.1
Gamma (P.1)	T1027I	MZ169911.1
Delta (B.1.617.2)	D950N	MZ359841.1
Omicron (B.1.1.529: BA.1)	Q954H, N969K, L981F	OL672836.1
Omicron (B.1.1.529: BA.2, BA.4, BA.5)	Q954H, N969K	OM685375.1 (BA.2)<br>ON373214.1 (BA.4)<br>ON249995.1 (BA.5)
**Variants of interest**		
**PANGO Lineage**	**Mutations**	**GenBank Reference**
Epsilon (B.1.427, B.1.429)	-	MW453103.1
Zeta (P.2)	-	MW523796.1
Eta (B.1.525)	F888L	MW560924.1
Iota (B.1.526)	-	MW643362.1
Kappa (B.1.617.1)	-	MW966601.1
Lambda (C.37)	-	MW850639.1
Mu (B.1.621)	D950N	EPI_ISL_1220045 (GISAID)

Table S2. Primers used for PCR-based site directed mutagenesis (QuikChange) to generate mutations. The plasmid backbone used was attB-S-mNG2-11.

Primer	Sequence (5′ to 3′)
T961F-F	GCACTGAACTTCCTGGTCAAGCAGCTGTCC
T961F-R	CAGCTGCTTGACCAGGAAGTTCAGTGCCTGGGC
K986P-F	CTGAGCAGACTGGACCCGGTGGAAGCCGAGGTGCAG
K986P-R	CTGCACCTCGGCTTCCACCGGGTCCAGTCTGCTCAG
V987P-F	CTGAGCAGACTGGACAAGCCGGAAGCCGAGGTGCAG
V987P-R	CTGCACCTCGGCTTCCGGCTTGTCCAGTCTGCTCAG
D994E-F	CGAGGTGCAGATCGAGAGACTGATCACCGGAAG
D994E-R	CTTCCGGTGATCAGTCTCTCGATCTGCACCTCGGCT
D994Q-F	GCCGAGGTGCAGATCCAGAGACTGATCACCGGAAG
D994Q-R	CTTCCGGTGATCAGTCTCTGGATCTGCACCTCGGCT
Q1005R-F	GGCTGCAGTCCCTGCGGACCTACGTTACCCAG
Q1005R-R	CTGGGTAACGTAGGTCCGCAGGGACTGCAGCC
K986P/V987P-F	CTGAGCAGACTGGACCCGCCGGAAGCCGAGGTGCAG
K986P/V987P-R	CTGCACCTCGGCTTCCGGCGGGTCCAGTCTGCTCAG

Table S3. Cassette primers to generate the DMS library.

Primer	Sequence (5′ to 3′)
Cassette1_1	GCCCTGCTGGCCGGCACAATCNNKAGTGGTTGGACATTTGGAGCTGGCGCCGCTCTGCAG
Cassette1_2	GCCCTGCTGGCCGGCACAATCACCNNKGGCTGGACCTTTGGAGCTGGCGCCGCTCTGCAG
Cassette1_3	GCCCTGCTGGCCGGCACAATCACCAGCNNKTGGACATTCGGAGCTGGCGCCGCTCTGCAG
Cassette1_4	GCCCTGCTGGCCGGCACAATCACAAGCGGTNNKACCTTTGGAGCTGGCGCCGCTCTGCAG
Cassette1_5	GCCCTGCTGGCCGGCACAATCACAAGTGGCTGGNNKTTCGGAGCTGGCGCCGCTCTGCAG
Cassette1_6	GCCCTGCTGGCCGGCACAATCACCAGCGGTTGGACCNNKGGTGCTGGCGCCGCTCTGCAG
Cassette1_7	GCCCTGCTGGCCGGCACAATCACCAGCGGTTGGACATTTNNKGCAGGCGCCGCTCTGCAG
Cassette1_8	GCCCTGCTGGCCGGCACAATCACCAGTGGTTGGACCTTCGGANNKGGCGCCGCTCTGCAG
Cassette2_1	AGCGGCTGGACATTTGGAGCTNNKGCAGCACTGCAGATCCCCTTTGCTATGCAGATGGCC
Cassette2_2	AGCGGCTGGACATTTGGAGCTGGTNNKGCTCTCCAGATCCCCTTTGCTATGCAGATGGCC
Cassette2_3	AGCGGCTGGACATTTGGAGCTGGTGCCNNKCTGCAAATCCCCTTTGCTATGCAGATGGCC
Cassette2_4	AGCGGCTGGACATTTGGAGCTGGCGCAGCTNNKCAAATCCCCTTTGCTATGCAGATGGCC
Cassette2_5	AGCGGCTGGACATTTGGAGCTGGCGCCGCACTCNNKATCCCCTTTGCTATGCAGATGGCC
Cassette2_6	AGCGGCTGGACATTTGGAGCTGGTGCAGCACTCCAANNKCCCTTTGCTATGCAGATGGCC
Cassette2_7	AGCGGCTGGACATTTGGAGCTGGTGCAGCTCTGCAAATANNKTTTGCTATGCAGATGGCC
Cassette2_8	AGCGGCTGGACATTTGGAGCTGGTGCCGCACTCCAGATACCCNNKGCTATGCAGATGGCC
Cassette3_1	GCCGCTCTGCAGATCCCCTTTNNKATGCAAATGGCATACCGGTTCAACGGCATCGGAGTG
Cassette3_2	GCCGCTCTGCAGATCCCCTTTGCANNKCAAATGGCCTATCGGTTCAACGGCATCGGAGTG
Cassette3_3	GCCGCTCTGCAGATCCCCTTTGCAATGNNKATGGCATATCGATTCAACGGCATCGGAGTG
Cassette3_4	GCCGCTCTGCAGATCCCCTTTGCTATGCAGNNKGCATATCGGTTCAACGGCATCGGAGTG
Cassette3_5	GCCGCTCTGCAGATCCCCTTTGCAATGCAAATGNNKTACCGATTTAACGGCATCGGAGTG

Cassette3_6	GCCGCTCTGCAGATCCCCTTTGCTATGCAAATGGCCNNKCGATTCAAC GGCATCGGAGTG
Cassette3_7	GCCGCTCTGCAGATCCCCTTTGCAATGCAGATGGCCTATNNKTTTAAC GGCATCGGAGTG
Cassette3_8	GCCGCTCTGCAGATCCCCTTTGCTATGCAGATGGCATACCGANNKAA CGGCATCGGAGTG
Cassette4_1	ATGCAGATGGCCTACCGGTTCNNKGGTATAGGAGTGACCCAGAATGT GCTGTACGAGAAC
Cassette4_2	ATGCAGATGGCCTACCGGTTCAATNNKATCGGTGTGACCCAGAATGT GCTGTACGAGAAC
Cassette4_3	ATGCAGATGGCCTACCGGTTCAACGGTNNKGGTGTAACCCAGAATGT GCTGTACGAGAAC
Cassette4_4	ATGCAGATGGCCTACCGGTTCAATGGCATANNKGTAACCCAGAATGT GCTGTACGAGAAC
Cassette4_5	ATGCAGATGGCCTACCGGTTCAACGGTATCGGANNKACACAGAATGT GCTGTACGAGAAC
Cassette4_6	ATGCAGATGGCCTACCGGTTCAACGGCATAGGTGTGNNKCAGAATGT GCTGTACGAGAAC
Cassette4_7	ATGCAGATGGCCTACCGGTTCAATGGTATCGGAGTAACCNNKAATGT GCTGTACGAGAAC
Cassette4_8	ATGCAGATGGCCTACCGGTTCAACGGCATCGGTGTAACACAGNNKGT GCTGTACGAGAAC
Cassette5_1	GGCATCGGAGTGACCCAGAATNNKCTCTATGAGAACCAGAAGCTGAT CGCCAACCAGTTC
Cassette5_2	GGCATCGGAGTGACCCAGAATGTANNKTACGAAAACCAGAAGCTGAT CGCCAACCAGTTC
Cassette5_3	GGCATCGGAGTGACCCAGAATGTACTGNNKGAGAATCAGAAGCTGAT CGCCAACCAGTTC
Cassette5_4	GGCATCGGAGTGACCCAGAATGTGCTCTACNNKAATCAGAAGCTGAT CGCCAACCAGTTC
Cassette5_5	GGCATCGGAGTGACCCAGAATGTGCTGTATGAANNKCAGAAGCTGAT CGCCAACCAGTTC
Cassette5_6	GGCATCGGAGTGACCCAGAATGTACTCTATGAAAATNNKAAGCTGATC GCCAACCAGTTC
Cassette5_7	GGCATCGGAGTGACCCAGAATGTACTCTACGAGAACCAANNKCTGAT CGCCAACCAGTTC
Cassette5_8	GGCATCGGAGTGACCCAGAATGTACTGTATGAGAACCAAAAGNNKAT CGCCAACCAGTTC
Cassette6_1	CTGTACGAGAACCAGAAGCTGNNKGCAAATCAGTTCAACAGCGCCAT CGGCAAGATCCAG
Cassette6_2	CTGTACGAGAACCAGAAGCTGATANNKAACCAATTCAACAGCGCCATC GGCAAGATCCAG
Cassette6_3	CTGTACGAGAACCAGAAGCTGATCGCANNKCAATTTAACAGCGCCATC GGCAAGATCCAG

Cassette6_4	CTGTACGAGAACCAGAAGCTGATAGCCAATNNKTTTAACAGCGCCATC GGCAAGATCCAG
Cassette6_5	CTGTACGAGAACCAGAAGCTGATCGCAAACCAGNNKAATAGCGCCAT CGGCAAGATCCAG
Cassette6_6	CTGTACGAGAACCAGAAGCTGATCGCCAATCAATTCNNKAGCGCCAT CGGCAAGATCCAG
Cassette6_7	CTGTACGAGAACCAGAAGCTGATAGCAAACCAGTTTAACNNKGCCATC GGCAAGATCCAG
Cassette6_8	CTGTACGAGAACCAGAAGCTGATCGCCAACCAATTTAATAGCNNKATC GGCAAGATCCAG
Cassette7_1	GCCAACCAGTTCAACAGCGCCNNKGGTAAAATCCAGGACAGCCTGAG CAGCACAGCAAGC
Cassette7_2	GCCAACCAGTTCAACAGCGCCATANNKAAGATACAGGACAGCCTGAG CAGCACAGCAAGC
Cassette7_3	GCCAACCAGTTCAACAGCGCCATCGGTNNKATACAAGACAGCCTGAG CAGCACAGCAAGC
Cassette7_4	GCCAACCAGTTCAACAGCGCCATAGGCAAANNKCAAGACAGCCTGAG CAGCACAGCAAGC
Cassette7_5	GCCAACCAGTTCAACAGCGCCATCGGTAAGATCNNKGATAGCCTGAG CAGCACAGCAAGC
Cassette7_6	GCCAACCAGTTCAACAGCGCCATCGGCAAAATACAGNNKAGCCTGAG CAGCACAGCAAGC
Cassette7_7	GCCAACCAGTTCAACAGCGCCATAGGTAAGATCCAAGACNNKCTGAG CAGCACAGCAAGC
Cassette7_8	GCCAACCAGTTCAACAGCGCCATCGGCAAGATACAAGATAGCNNKAG CAGCACAGCAAGC
Cassette8_1	GGCAAGATCCAGGACAGCCTGNNKAGTACCGCAAGCGCCCTGGGAA AGCTGCAGGACGTG
Cassette8_2	GGCAAGATCCAGGACAGCCTGAGTNNKACAGCCAGCGCCCTGGGAA AGCTGCAGGACGTG
Cassette8_3	GGCAAGATCCAGGACAGCCTGAGCAGCNNKGCCAGTGCCCTGGGAA AGCTGCAGGACGTG
Cassette8_4	GGCAAGATCCAGGACAGCCTGAGCAGCACCNNKAGCGCACTGGGAA AGCTGCAGGACGTG
Cassette8_5	GGCAAGATCCAGGACAGCCTGAGCAGTACAGCANNKGCACTGGGAAA GCTGCAGGACGTG
Cassette8_6	GGCAAGATCCAGGACAGCCTGAGTAGCACCGCAAGTNNKCTGGGAAA GCTGCAGGACGTG
Cassette8_7	GGCAAGATCCAGGACAGCCTGAGTAGTACCGCCAGTGCCNNKGGAAA GCTGCAGGACGTG
Cassette8_8	GGCAAGATCCAGGACAGCCTGAGTAGTACAGCAAGTGCCCTGNNKAA GCTGCAGGACGTG
Cassette9_1	AGCACAGCAAGCGCCCTGGGANNKCTCCAAGACGTGGTCAACCAGAA TGCCCAGGCACTG

Cassette9_2	AGCACAGCAAGCGCCCTGGGAAAANNKCAGGATGTGGTCAACCAGAA TGCCCAGGCACTG
Cassette9_3	AGCACAGCAAGCGCCCTGGGAAAGCTCNNKGATGTAGTCAACCAGAA TGCCCAGGCACTG
Cassette9_4	AGCACAGCAAGCGCCCTGGGAAAACTGCAANNKGTAGTCAACCAGAA TGCCCAGGCACTG
Cassette9_5	AGCACAGCAAGCGCCCTGGGAAAGCTCCAGGACNNKGTAAACCAGAA TGCCCAGGCACTG
Cassette9_6	AGCACAGCAAGCGCCCTGGGAAAGCTGCAAGATGTGNNKAACCAGAA TGCCCAGGCACTG
Cassette9_7	AGCACAGCAAGCGCCCTGGGAAAACTCCAGGACGTAGTCNNKCAGAA TGCCCAGGCACTG
Cassette9_8	AGCACAGCAAGCGCCCTGGGAAAGCTGCAAGACGTAGTAAACNNKAA TGCCCAGGCACTG
Cassette10_1	CTGCAGGACGTGGTCAACCAGNNKGCACAAGCACTGAACACCCTGGT CAAGCAGCTGTCC
Cassette10_2	CTGCAGGACGTGGTCAACCAGAACNNKCAGGCCCTGAACACCCTGGT CAAGCAGCTGTCC
Cassette10_3	CTGCAGGACGTGGTCAACCAGAACGCCNNKGCACTCAACACCCTGGT CAAGCAGCTGTCC
Cassette10_4	CTGCAGGACGTGGTCAACCAGAATGCACAGNNKCTCAACACCCTGGT CAAGCAGCTGTCC
Cassette10_5	CTGCAGGACGTGGTCAACCAGAATGCCCAAGCCNNKAACACCCTGGT CAAGCAGCTGTCC
Cassette10_6	CTGCAGGACGTGGTCAACCAGAACGCACAAGCCCTCNNKACCCTGGT CAAGCAGCTGTCC
Cassette10_7	CTGCAGGACGTGGTCAACCAGAACGCACAGGCACTCAATNNKCTGGT CAAGCAGCTGTCC
Cassette10_8	CTGCAGGACGTGGTCAACCAGAACGCCCAAGCACTGAATACCNNKGT CAAGCAGCTGTCC
Cassette11_1	GCCCAGGCACTGAACACCCTGNNKAAACAACTGTCCTCCAACTTCGG CGCCATCAGCTCT
Cassette11_2	GCCCAGGCACTGAACACCCTGGTANNKCAGCTCTCCTCCAACTTCGG CGCCATCAGCTCT
Cassette11_3	GCCCAGGCACTGAACACCCTGGTAAAGNNKCTGTCTTCCAACTTCGG CGCCATCAGCTCT
Cassette11_4	GCCCAGGCACTGAACACCCTGGTCAAACAGNNKTCTTCCAACTTCGG CGCCATCAGCTCT
Cassette11_5	GCCCAGGCACTGAACACCCTGGTCAAGCAACTCNNKTCCAACTTCGG CGCCATCAGCTCT
Cassette11_6	GCCCAGGCACTGAACACCCTGGTAAAACAACTCTCTNNKAACTTCGG CGCCATCAGCTCT
Cassette11_7	GCCCAGGCACTGAACACCCTGGTAAAGCAACTCTCCTCTNNKTTCGG CGCCATCAGCTCT

Cassette11_8	GCCCAGGCACTGAACACCCTGGTAAAGCAGCTCTCTTCTAACNNKGG CGCCATCAGCTCT
Cassette12_1	AAGCAGCTGTCCTCCAACTTCNNKGCAATAAGCTCTGTGCTGAACGAT ATCCTGAGCAGA
Cassette12_2	AAGCAGCTGTCCTCCAACTTCGGTNNKATCAGTTCTGTGCTGAACGAT ATCCTGAGCAGA
Cassette12_3	AAGCAGCTGTCCTCCAACTTCGGTGCCNNKAGCTCGGTGCTGAACGA TATCCTGAGCAGA
Cassette12_4	AAGCAGCTGTCCTCCAACTTCGGCGCAATCNNKTCGGTGCTGAACGA TATCCTGAGCAGA
Cassette12_5	AAGCAGCTGTCCTCCAACTTCGGCGCCATAAGTNNKGTGCTGAACGA TATCCTGAGCAGA
Cassette12_6	AAGCAGCTGTCCTCCAACTTCGGTGCAATAAGTTCGNNKCTGAACGAT ATCCTGAGCAGA
Cassette12_7	AAGCAGCTGTCCTCCAACTTCGGTGCAATCAGCTCTGTANNKAACGAT ATCCTGAGCAGA
Cassette12_8	AAGCAGCTGTCCTCCAACTTCGGTGCCATCAGTTCGGTACTGNNKGAT ATCCTGAGCAGA
Cassette13_1	GCCATCAGCTCTGTGCTGAACNNKATACTCAGCAGACTGGACAAGGT GGAAGCCGAGGTG
Cassette13_2	GCCATCAGCTCTGTGCTGAACGACNNKCTGAGTAGACTGGACAAGGT GGAAGCCGAGGTG
Cassette13_3	GCCATCAGCTCTGTGCTGAACGATATCNNKAGTAGGCTGGACAAGGT GGAAGCCGAGGTG
Cassette13_4	GCCATCAGCTCTGTGCTGAACGATATCCTCNNKAGACTCGACAAGGT GGAAGCCGAGGTG
Cassette13_5	GCCATCAGCTCTGTGCTGAACGATATACTGAGCNNKCTCGACAAGGT GGAAGCCGAGGTG
Cassette13_6	GCCATCAGCTCTGTGCTGAACGACATCCTCAGCAGGNNKGACAAGGT GGAAGCCGAGGTG
Cassette13_7	GCCATCAGCTCTGTGCTGAACGACATACTCAGTAGGCTGNNKAAGGT GGAAGCCGAGGTG
Cassette13_8	GCCATCAGCTCTGTGCTGAACGACATACTGAGCAGGCTGGACNNKGT GGAAGCCGAGGTG
Cassette14_1	ATCCTGAGCAGACTGGACAAGNNKGAGGCAGAGGTGCAGATCGACA GACTGATCACCGGA
Cassette14_2	ATCCTGAGCAGACTGGACAAGGTANNKGCCGAAGTGCAGATCGACAG ACTGATCACCGGA
Cassette14_3	ATCCTGAGCAGACTGGACAAGGTAGAANNKGAGGTACAGATCGACAG ACTGATCACCGGA
Cassette14_4	ATCCTGAGCAGACTGGACAAGGTGGAGGCCNNKGTACAGATCGACAG ACTGATCACCGGA
Cassette14_5	ATCCTGAGCAGACTGGACAAGGTGGAAGCAGAANNKCAGATCGACAG ACTGATCACCGGA

Cassette14_6	ATCCTGAGCAGACTGGACAAGGTAGAGGCAGAAGTANNKATCGACAG ACTGATCACCGGA
Cassette14_7	ATCCTGAGCAGACTGGACAAGGTAGAGGCCGAGGTACAANNKGACAG ACTGATCACCGGA
Cassette14_8	ATCCTGAGCAGACTGGACAAGGTAGAAGCAGAGGTGCAAATCNNKAG ACTGATCACCGGA
Cassette15_1	GAAGCCGAGGTGCAGATCGACNNKCTCATAACCGGAAGGCTGCAGTC CCTGCAGACCTAC
Cassette15_2	GAAGCCGAGGTGCAGATCGACAGGNNKATCACAGGAAGGCTGCAGT CCCTGCAGACCTAC
Cassette15_3	GAAGCCGAGGTGCAGATCGACAGGCTGNNKACCGGTAGGCTGCAGT CCCTGCAGACCTAC
Cassette15_4	GAAGCCGAGGTGCAGATCGACAGACTCATCNNKGGTAGGCTGCAGTC CCTGCAGACCTAC
Cassette15_5	GAAGCCGAGGTGCAGATCGACAGACTGATAACANNKAGGCTGCAGTC CCTGCAGACCTAC
Cassette15_6	GAAGCCGAGGTGCAGATCGACAGGCTCATAACAGGTNNKCTGCAGTC CCTGCAGACCTAC
Cassette15_7	GAAGCCGAGGTGCAGATCGACAGGCTCATCACCGGTAGANNKCAGTC CCTGCAGACCTAC
Cassette15_8	GAAGCCGAGGTGCAGATCGACAGGCTGATCACCGGAAGACTGNNKTC CCTGCAGACCTAC
Cassette16_1	CTGATCACCGGAAGGCTGCAGNNKCTCCAAACCTACGTTACCCAGCA GCTGATCAGAGCC
Cassette16_2	CTGATCACCGGAAGGCTGCAGTCTNNKCAGACATACGTTACCCAGCA GCTGATCAGAGCC
Cassette16_3	CTGATCACCGGAAGGCTGCAGTCTCTGNNKACCTATGTTACCCAGCA GCTGATCAGAGCC
Cassette16_4	CTGATCACCGGAAGGCTGCAGTCCCTCCAGNNKTATGTTACCCAGCA GCTGATCAGAGCC
Cassette16_5	CTGATCACCGGAAGGCTGCAGTCCCTGCAAACANNKGTTACCCAGCA GCTGATCAGAGCC
Cassette16_6	CTGATCACCGGAAGGCTGCAGTCTCTCCAAACATATNNKACCCAGCA GCTGATCAGAGCC
Cassette16_7	CTGATCACCGGAAGGCTGCAGTCTCTCCAGACCTATGTANNKCAGCA GCTGATCAGAGCC
Cassette16_8	CTGATCACCGGAAGGCTGCAGTCTCTGCAAACCTACGTAACCNNKCA GCTGATCAGAGCC
Cassette17_1	CTGCAGACCTACGTTACCCAGNNKCTCATAAGAGCCGCCGAGATTAG AGCCTCTGCCAAT
Cassette17_2	CTGCAGACCTACGTTACCCAGCAANNKATCAGGGCCGCCGAGATTAG AGCCTCTGCCAAT
Cassette17_3	CTGCAGACCTACGTTACCCAGCAACTGNNKAGAGCAGCCGAGATTAG AGCCTCTGCCAAT

Cassette17_4	CTGCAGACCTACGTTACCCAGCAGCTCATCNNKGCAGCCGAGATTAG AGCCTCTGCCAAT
Cassette17_5	CTGCAGACCTACGTTACCCAGCAGCTGATAAGGNNKGCCGAGATTAG AGCCTCTGCCAAT
Cassette17_6	CTGCAGACCTACGTTACCCAGCAACTCATAAGGGCANNKGAGATTAG AGCCTCTGCCAAT
Cassette17_7	CTGCAGACCTACGTTACCCAGCAACTCATCAGAGCAGCANNKATTAGA GCCTCTGCCAAT
Cassette17_8	CTGCAGACCTACGTTACCCAGCAACTGATCAGGGCAGCAGAGNNKAG AGCCTCTGCCAAT
Cassette18_1	CTGATCAGAGCCGCCGAGATTNNKGCATCGGCCAATCTGGCCGCCAC CAAGATGTCTGAG
Cassette18_2	CTGATCAGAGCCGCCGAGATTAGGNNKTCTGCAAATCTGGCCGCCAC CAAGATGTCTGAG
Cassette18_3	CTGATCAGAGCCGCCGAGATTAGGGCCNNKGCCAACCTGGCCGCCA CCAAGATGTCTGAG
Cassette18_4	CTGATCAGAGCCGCCGAGATTAGAGCATCTNNKAACCTGGCCGCCAC CAAGATGTCTGAG
Cassette18_5	CTGATCAGAGCCGCCGAGATTAGAGCCTCGGCANNKCTGGCCGCCA CCAAGATGTCTGAG
Cassette18_6	CTGATCAGAGCCGCCGAGATTAGGGCATCGGCAAACNNKGCCGCCA CCAAGATGTCTGAG
Cassette18_7	CTGATCAGAGCCGCCGAGATTAGGGCATCTGCCAACCTCNNKGCCAC CAAGATGTCTGAG
Cassette18_8	CTGATCAGAGCCGCCGAGATTAGGGCCTCTGCAAACCTCGCCNNKAC CAAGATGTCTGAG
Cassette19_1	GCCTCTGCCAATCTGGCCGCCNNKAAAATGTCGGAGTGTGTGCTGGG CCAGAGCAAGAGA
Cassette19_2	GCCTCTGCCAATCTGGCCGCCACANNKATGTCTGAATGTGTGCTGGG CCAGAGCAAGAGA
Cassette19_3	GCCTCTGCCAATCTGGCCGCCACCAAGNNKTCGGAATGTGTGCTGGG CCAGAGCAAGAGA
Cassette19_4	GCCTCTGCCAATCTGGCCGCCACCAAAATGNNKGAATGCGTGCTGGG CCAGAGCAAGAGA
Cassette19_5	GCCTCTGCCAATCTGGCCGCCACAAAGATGTCGNNKTGCGTGCTGGG CCAGAGCAAGAGA
Cassette19_6	GCCTCTGCCAATCTGGCCGCCACCAAGATGTCTGAANNKGTACTGGG CCAGAGCAAGAGA
Cassette19_7	GCCTCTGCCAATCTGGCCGCCACAAAAATGTCTGAGTGCNNKCTGGG CCAGAGCAAGAGA
Cassette19_8	GCCTCTGCCAATCTGGCCGCCACCAAGATGTCGGAGTGTGTANNKGG CCAGAGCAAGAGA

Cassette1_Rprimer	GATTGTGCCGGCCAGCAGGGC
Cassette2_Rprimer	AGCTCCAAATGTCCAGCCGCT
Cassette3_Rprimer	AAAGGGGATCTGCAGAGCGGC
Cassette4_Rprimer	GAACCGGTAGGCCATCTGCAT
Cassette5_Rprimer	ATTCTGGGTCACTCCGATGCC
Cassette6_Rprimer	CAGCTTCTGGTTCTCGTACAG
Cassette7_Rprimer	GGCGCTGTTGAACTGGTTGGC
Cassette8_Rprimer	CAGGCTGTCCTGGATCTTGCC
Cassette9_Rprimer	TCCCAGGGCGCTTGCTGTGCT
Cassette10_Rprimer	CTGGTTGACCACGTCCTGCAG
Cassette11_Rprimer	CAGGGTGTTCAGTGCCTGGGC
Cassette12_Rprimer	GAAGTTGGAGGACAGCTGCTT
Cassette13_Rprimer	GTTCAGCACAGAGCTGATGGC
Cassette14_Rprimer	CTTGTCCAGTCTGCTCAGGAT
Cassette15_Rprimer	GTCGATCTGCACCTCGGCTTC
Cassette16_Rprimer	CTGCAGCCTTCCGGTGATCAG
Cassette17_Rprimer	CTGGGTAACGTAGGTCTGCAG
Cassette18_Rprimer	AATCTCGGCGGCTCTGATCAG
Cassette19_Rprimer	GGCGGCCAGATTGGCAGAGGC

Table S4. Numbers of cells collected per bin in expression sorting.

Bin	Replicate 1	Replicate 2	Replicate 3
Bin 0	8.03×10^5	1.30×10^6	1.70×10^6
Bin 1	7.51×10^5	1.30×10^6	1.70×10^6
Bin 2	7.70×10^5	1.30×10^6	1.70×10^6
Bin 3	8.20×10^5	1.30×10^6	1.70×10^6

Table S5. Numbers of cells collected per bin in fusion sorting.

Bin	Replicate 1	Replicate 2
$mNG2^-$	3.53×10^6	5.51×10^6
$mNG2^+$	1.84×10^5	4.87×10^5

Table S6. *p*-values from Student's *t* test of expression and fusion scores between mutation types.

**Expression**			
	Missense	Nonsense	Silent
Missense		6.46×10^{-60}	2.93×10^{-2}
Nonsense	6.46×10^{-60}		3.80×10^{-44}
Silent	2.93×10^{-2}	3.80×10^{-44}	
**Fusion**			
	Missense	Nonsense	Silent
Missense		3.10×10^{-34}	7.08×10^{-4}
Nonsense	3.10×10^{-34}		1.44×10^{-31}
Silent	7.08×10^{-4}	1.44×10^{-31}	

Table S7. Betacoronaviruses used for sequence conservation analysis.

Accession ID	Database	Name
gb_MN908947.3_	GenBank	Severe acute respiratory syndrome coronavirus 2 isolate Wuhan-Hu-1, complete genome
gb_MN996532.2_	GenBank	Bat coronavirus RaTG13, complete genome
gb_MZ937000.1_	GenBank	Bat coronavirus isolate BANAL-20-52/Laos/2020, complete genome
EPI_ISL_410543	GISAID	hCoV-19/pangolin/Guangxi/P3B/2017
EPI_ISL_471465	GISAID	hCoV-19/pangolin/Guangdong/cDNA20-S/2019
gb_MZ937003.1_	GenBank	Bat coronavirus isolate BANAL-20-236/Laos/2020, complete genome
gb_MZ937001.1_	GenBank	Bat coronavirus isolate BANAL-20-103/Laos/2020, complete genome
gb_MG772933.1_	GenBank	Bat SARS-like coronavirus isolate bat-SL-CoVZC45, complete genome
gb_MG772934.1_	GenBank	Bat SARS-like coronavirus isolate bat-SL-CoVZXC21, complete genome
gb_KT444582.1_	GenBank	SARS-like coronavirus WIV16, complete genome
gb_DQ497008.1_	GenBank	SARS coronavirus strain MA-15, complete genome
gb_KC881007.1_	GenBank	Bat SARS-like coronavirus WIV1 spike protein (S) gene, complete cds
gb_KY417144.1_	GenBank	Bat SARS-like coronavirus isolate Rs4084, complete genome
gb_DQ412042.1_	GenBank	Bat SARS coronavirus Rf1, complete genome
gb_KJ473813.1_	GenBank	BtRf-BetaCoV/SX2013, complete genome
gb_KJ473815.1_	GenBank	BtRs-BetaCoV/GX2013, complete genome
gb_KF294457.1_	GenBank	Bat SARS-like coronavirus isolate Longquan-140 orf1ab polyprotein, spike glycoprotein, envelope protein, membrane protein, and nucleocapsid protein genes, complete cds
gb_DQ022305.2_	GenBank	Bat SARS coronavirus HKU3-1, complete genome
gb_DQ071615.1_	GenBank	Bat SARS coronavirus Rp3, complete genome
gb_FJ588686.1_	GenBank	Bat SARS CoV Rs672/2006, complete genome
gb_KJ473814.1_	GenBank	BtRs-BetaCoV/HuB2013, complete genome
gb_KF569996.1_	GenBank	*Rhinolophus affinis* coronavirus isolate LYRa11, complete genome
gb_KY352407.1_	GenBank	Severe acute respiratory syndrome-related coronavirus strain BtKY72, complete genome
ref_NC_014470.1_	GenBank	Bat coronavirus BM48-31/BGR/2008, complete genome
gb_MZ937004.1_	GenBank	Bat coronavirus isolate BANAL-20-247/Laos/2020, complete genome
gb_MZ937002.1_	GenBank	Bat coronavirus isolate BANAL-20-116/Laos/2020, complete genome

EPI_ISL_412977	GISAID	hCoV-19/bat/Yunnan/RmYN02/2019

Table S8. Cryo-EM data collection statistics.

	2PQ spike (EMDB-29374)
**Data collection and processing**	
Magnification	130,000
Voltage (kV)	300
Electron exposure (e^-/Å^2)	50
Defocus range (μm)	-0.8 to -1.5
Pixel size (Å)	0.66
Symmetry imposed	C1
Initial particle images (no.)	238,524
Final particle images (no.)	140,183
Map resolution (Å)	3.66
FSC threshold	0.143
Map resolution range (Å)	N/A

SUPPLEMENTARY REFERENCES

1. Zhou, P. *et al.* A human antibody reveals a conserved site on beta-coronavirus spike proteins and confers protection against SARS-CoV-2 infection. *Sci. Transl. Med.* **14**, eabi9215 (2022).

2. Walls, A. C. *et al.* Structure, function, and antigenicity of the SARS-CoV-2 spike glycoprotein. *Cell* **181**, 281-292. e6 (2020).

3. McCallum, M. *et al.* N-terminal domain antigenic mapping reveals a site of vulnerability for SARS-CoV-2. *Cell* **184**, 2332-2347.e16 (2021).

4. Yuan, M. *et al.* Structural basis of a shared antibody response to SARS-CoV-2. *Science* **369**, 1119-1123 (2020).

5. Claireaux, M. *et al.* A public antibody class recognizes an S2 epitope exposed on open conformations of SARS-CoV-2 spike. *Nat. Commun.* **13**, 4539 (2022).
