# Supplementary material for: High-throughput identification of prefusion-stabilizing mutations in SARS-CoV-2 spike
Source: Nat Commun. 2023 Apr 10;14:2003. doi: 10.1038/s41467-023-37786-1 (PMC10086000; doi:10.1038/s41467-023-37786-1)
Supplement: Supplementary file 2 — Reporting Summary [file 41467_2023_37786_MOESM2_ESM.pdf]

nature portfolio

Corresponding author(s): Nicholas Wu

Last updated by author(s): March 22, 2023

# Reporting Summary

Nature Portfolio wishes to improve the reproducibility of the work that we publish. This form provides structure for consistency and transparency in reporting. For further information on Nature Portfolio policies, see our Editorial Policies and the Editorial Policy Checklist.

## Statistics

For all statistical analyses, confirm that the following items are present in the figure legend, table legend, main text, or Methods section.

| n/a | Confirmed | |
|---|---|---|
| [ ] | [x] | The exact sample size ($n$) for each experimental group/condition, given as a discrete number and unit of measurement |
| [ ] | [x] | A statement on whether measurements were taken from distinct samples or whether the same sample was measured repeatedly |
| [ ] | [x] | The statistical test(s) used AND whether they are one- or two-sided<br>*Only common tests should be described solely by name; describe more complex techniques in the Methods section.* |
| [x] | [ ] | A description of all covariates tested |
| [x] | [ ] | A description of any assumptions or corrections, such as tests of normality and adjustment for multiple comparisons |
| [ ] | [x] | A full description of the statistical parameters including central tendency (e.g. means) or other basic estimates (e.g. regression coefficient) AND variation (e.g. standard deviation) or associated estimates of uncertainty (e.g. confidence intervals) |
| [ ] | [x] | For null hypothesis testing, the test statistic (e.g. $F$, $t$, $r$) with confidence intervals, effect sizes, degrees of freedom and $P$ value noted<br>*Give P values as exact values whenever suitable.* |
| [x] | [ ] | For Bayesian analysis, information on the choice of priors and Markov chain Monte Carlo settings |
| [x] | [ ] | For hierarchical and complex designs, identification of the appropriate level for tests and full reporting of outcomes |
| [ ] | [x] | Estimates of effect sizes (e.g. Cohen's $d$, Pearson's $r$), indicating how they were calculated |

*Our web collection on statistics for biologists contains articles on many of the points above.*

## Software and code

Policy information about availability of computer code

| | |
|---|---|
| Data collection | For flow cytometry analysis using the BD Accuri C6 cytometer, data was collected using BD Accuri C6 software v264 (BD Biosciences).<br>For sorting using the BD FACSAria II cell sorter, data was collected using BD FACSDiva software v8.0 (BD Biosciences).<br>For sorting the Bigfoot spectral cell sorter, data was collected using Sasquatch software firmware v888 (Invitrogen).<br>For size-exclusion chromatography, data was collected using ChromLab software v6.1 (Bio-Rad).<br>For cryogenic electron microscopy, data was collected using serialEM v4.0.<br>For visualization of proteins, UCSF ChimeraX v1.5 (UCSF) or PyMOL v2.4.0 (Schrodinger) was used.<br>For in silico mutagenesis, Rosetta v3.11 (Rosetta Commons) was used. |
| Data analysis | Flow cytometry and fluorescence-activated cell sorting data were plotted and analyzed in FCS Express v6 (De Novo Software).<br>Cryogenic electron microscopy data processing was performed on-the-fly with cryoSPARC Live v3.3.2 (Structura Biotechnology).<br>Custom code has been deposited to https://github.com/nicwulab/SARS2_S_fusogenicity_DMS.<br>For plotting data, RStudio v2022.12.0+353 was used. |

For manuscripts utilizing custom algorithms or software that are central to the research but not yet described in published literature, software must be made available to editors and reviewers. We strongly encourage code deposition in a community repository (e.g. GitHub). See the Nature Portfolio guidelines for submitting code & software for further information.

## Data

Policy information about availability of data

All manuscripts must include a data availability statement. This statement should provide the following information, where applicable:

- Accession codes, unique identifiers, or web links for publicly available datasets
- A description of any restrictions on data availability
- For clinical datasets or third party data, please ensure that the statement adheres to our policy

Structures from the following identifiers from the Protein Data Bank (PDB) were used in this study: 6VXX [https://www.rcsb.org/structure/6vxx] and 6VYB [https://www.rcsb.org/structure/6vyb]. The cryoEM map of 2PQ spike can be accessed at the Electron Microscopy Data Bank (EMDB) using accession code EMD-29374 [https://www.ebi.ac.uk/emdb/EMD-29374]. Raw deep sequencing data generated in this study have been submitted to the NIH Sequence Read Archive under accession number: PRJNA826665 [https://www.ncbi.nlm.nih.gov/bioproject/PRJNA826665/]. Source data are provided with this paper.

## Human research participants

Policy information about studies involving human research participants and Sex and Gender in Research.

| | |
|---|---|
| Reporting on sex and gender | Not applicable in this study |
| Population characteristics | Not applicable in this study |
| Recruitment | Not applicable in this study |
| Ethics oversight | Not applicable in this study |

Note that full information on the approval of the study protocol must also be provided in the manuscript.

# Field-specific reporting

Please select the one below that is the best fit for your research. If you are not sure, read the appropriate sections before making your selection.

☒ Life sciences ☐ Behavioural & social sciences ☐ Ecological, evolutionary & environmental sciences

For a reference copy of the document with all sections, see nature.com/documents/nr-reporting-summary-flat.pdf

# Life sciences study design

All studies must disclose on these points even when the disclosure is negative.

| | |
|---|---|
| Sample size | In the context of this study, sample size refers to the number of amino acids in SARS-CoV-2 spike protein to be mutated. No sample size calculation was performed. We selected the entire region spanning the first heptad repeat and central helix of SARS-CoV-2 spike protein (152 amino acids long, see Methods) for our deep mutational scanning experiments. For our study, this is sufficient because this is the region primarily responsible for the transition from prefusion state to the postfusion conformation of the spike protein. |
| Data exclusions | No data were excluded in this study. |
| Replication | Two to three independent replicates were performed as indicated in the figure legends. |
| Randomization | Randomization is not relevant because we systematically screened for all amino acid mutations in the region covering the first heptad repeat and the central helix regions of the S2 subunit of SARS-CoV-2 spike. Mutations were classified as prefusion-stabilizing or fusion-competent based on fusion and expression scores (Fig. 2, see Methods). |
| Blinding | Blinding is not relevant because we systematically screened for all amino acid mutations in the region covering the first heptad repeat and the central helix regions of the S2 subunit of SARS-CoV-2 spike. |

# Reporting for specific materials, systems and methods

We require information from authors about some types of materials, experimental systems and methods used in many studies. Here, indicate whether each material, system or method listed is relevant to your study. If you are not sure if a list item applies to your research, read the appropriate section before selecting a response.

## Materials & experimental systems

| n/a | Involved in the study | |
|---|---|---|
| ☐ | ☒ | Antibodies |
| ☐ | ☒ | Eukaryotic cell lines |
| ☒ | ☐ | Palaeontology and archaeology |
| ☒ | ☐ | Animals and other organisms |
| ☒ | ☐ | Clinical data |
| ☒ | ☐ | Dual use research of concern |

## Methods

| n/a | Involved in the study | |
|---|---|---|
| ☒ | ☐ | ChIP-seq |
| ☐ | ☒ | Flow cytometry |
| ☒ | ☐ | MRI-based neuroimaging |

# Antibodies

Antibodies used

Primary antibodies (all in human IgG1 Fc format):
CC12.3 (PMID: 32661058; source: Meng Yuan, Ian A. Wilson)
CC40.8 (PMID: 35133175; source: Ge Song, Raiees Andrabi)
COVA1-07 (PMID: 35927266; source: made in-house using Expi293F cells)
S2M28 (PMID: 33773105; source: made in-house using Expi293F cells)

Secondary antibody:
PE anti-human IgG Fc, BioLegend, Cat. No. 410708, Clone M1310G05

Validation

Validation of epitopes of primary antibodies was performed using cryogenic electron microscopy by authors in their corresponding publications:
CC12.3 (PMID: 32661058)
CC40.8 (PMID: 35133175)
COVA1-07 (PMID: 35927266)
S2M28 (PMID: 33773105)

Validation of secondary antibody was performed by the manufacturer and written in their website (https://www.biolegend.com/nl-nl/products/pe-anti-human-igg-fc-11933).

# Eukaryotic cell lines

Policy information about cell lines and Sex and Gender in Research

Cell line source(s)

HEK293T landing pad cells were obtained from Dr Kenneth Matreyek (Case Western Reserve University).
Expi293F cells were obtained from Gibco and immediately used as directed.

Authentication

All cell lines were used as received. Cell lines were not authenticated.

Mycoplasma contamination

Untransfected and recombined HEK293T landing pad cells tested negative for mycoplasma contamination via staining with DAPI and imaging on a fluorescence microscope. Expi293F cells were not tested for mycoplasma contamination.

Commonly misidentified lines
(See ICLAC register)

No commonly misidentified cell lines were used in this study.

# Flow Cytometry

## Plots

Confirm that:

☒ The axis labels state the marker and fluorochrome used (e.g. CD4-FITC).

☒ The axis scales are clearly visible. Include numbers along axes only for bottom left plot of group (a 'group' is an analysis of identical markers).

☒ All plots are contour plots with outliers or pseudocolor plots.

☒ A numerical value for number of cells or percentage (with statistics) is provided.

## Methodology

Sample preparation

For expression assays, S-expressing cells were harvested and then resuspended in ice-cold FACS buffer (2% v/v FBS, 5 mM EDTA in DMEM without phenol red). Primary antibody used was 5 ug/mL CC12.3 and secondary antibody used was 1 ug/mL PE anti-human IgG Fc. Antibody staining was performed at 4 C for 1 h with gentle rocking. After washing with ice-cold FACS buffer, cells were filtered through a 40 um filter, and processed via flow cytometry or sorted.
For fusion assays, hACE2- and S-expressing cells were harvested, resuspended in complete growth medium and filtered through a 40 um filter. Cells were mixed at equal numbers to a total of 500,000 cells per mL of growth medium. Co-cultures were incubated for 3 h at 37 C and 5% CO2. Then, cells were harvested and resuspended in ice-cold FACS buffer. Cells were

then processed via flow cytometry or sorted.

| | |
|---|---|
| Instrument | For flow cytometry, cells were processed using a BD Accuri C6 cytometer (BD Biosciences).<br>For cell sorting, cells were sorted using either a BD FACSAria II cell sorter (BD Biosciences) or a Bigfoot spectral cell sorter (Invitrogen). |
| Software | For flow cytometry analysis using the BD Accuri C6 cytometer, data was collected using BD Accuri C6 software v264 (BD Biosciences).<br>For sorting using the BD FACSAria II cell sorter, data was collected using BD FACSDiva software v8.0 (BD Biosciences).<br>For sorting the Bigfoot spectral cell sorter, data was collected using Sasquatch software (Invitrogen).<br>All data were plotted and analyzed in FCS Express v6 (De Novo Software). |
| Cell population abundance | Purity analysis of the samples post-sort was not performed because samples were immediately processed for genomic DNA extraction post-sorting. |
| Gating strategy | For expression analysis, cells were first gated based on FSC-A/SSC-A. Singlets were gated based on Width/FSC-A. Finally, the PE-positive fraction was gated based on FSC-A/PE. Gating strategy is provided in Supplementary Fig. 11a. Ten thousand singlets were processed.<br>For fusion analysis, cells were first gated based on FSC-A/SSC-A. The mNG2-positive fraction was gated based on FSC-A/mNG2. Gating strategy is provided in Supplementary Fig. 11b. Ten thousand cells were processed.<br>For expression sorting, cells were first gated based on FSC-A/SSC-A. Singlets were gated based on Width/FSC-A. Finally, the PE-positive fractions were gated based on FSC-A/PE. Gating strategy is provided in Supplementary Fig. 11c.<br>For fusion sorting, cells were first gated based on FSC-A/SSC-A. The mNG2-positive and negative fractions were gated based on FSC-A/mNG2. Gating strategy is provided in Supplementary Fig. 11d. |

☒ Tick this box to confirm that a figure exemplifying the gating strategy is provided in the Supplementary Information.
